# The application of the Risdon approach for mandibular condyle fractures

**DOI:** 10.1186/1471-2482-13-25

**Published:** 2013-07-06

**Authors:** Seung Min Nam, Jang Hyun Lee, Jun Hyuk Kim

**Affiliations:** 1 Department of Plastic and Reconstructive Surgery, Soonchunhyang University Bucheon Hospital, Soonchunhyang University College of Medicine, Bucheon, Korea; 2 Department of Plastic and Reconstructive Surgery, Hanyang University Guri Hospital, Hanyang University College of Medicine, Guri, Korea; 3 Department of Plastic and Reconstructive Surgery, Soonchunhyang University Cheonan Hospital, Soonchunhyang University College of Medicine, Cheonan, Korea

**Keywords:** Mandibular condyle, Mandibular injuries, Operative surgical procedure

## Abstract

**Background:**

Many novel approaches to mandibular condyle fracture have been reported, but there is a relative lack of reports on the Risdon approach. In this study, the feasibility of the Risdon approach for condylar neck and subcondylar fractures of the mandible is demonstrated.

**Methods:**

A review of patients with mandibular condylar neck and subcondylar fractures was performed from March 2008 to June 2012. A total of 25 patients, 19 males and 6 females, had 14 condylar neck fractures and 11 subcondylar fractures.

**Results:**

All of the cases were reduced using the Risdon approach. For subcondylar fractures, reduction and fixation with plates was done under direct vision. For condylar neck fractures, reduction and fixation was done with the aid of a trochar in adults and a percutaneous threaded Kirschner wire in children. There were no malunions or nonunions revealed in follow-up care. Mild transient neuropraxia of the marginal mandibular nerve was seen in 4 patients, which was resolved within 1–2 months.

**Conclusions:**

The Risdon approach is a technique for reducing the condylar neck and subcondylar fractures that is easy to perform and easy to learn. Its value in the reduction of mandibular condyle fractures should be emphasized.

## Background

Of all the fractures of the facial skeleton, the choice of treatment modalities for mandibular condyle fractures is probably the most controversial [1]. The controversy has regarded closed conservative management versus open surgical management of condylar and subcondylar fractures, which constitute 25-35% of all mandible fractures reported in the literature [2]. Those who prefer conservative treatment argue that morbidity due to surgical treatment is much greater than the advantages gained, and that 3–4 weeks of intermaxillary fixation and early mouth opening exercises are enough to achieve good results. Those who advocate surgical treatment argue that only definite open reduction can prevent shortening of the ramus, facial asymmetry, and ankylosis of the temporomandibular joint (TMJ), while providing a shortened time for the recovery of mastication and TMJ function [2-4].

For cases where open reduction is necessary, the surgeon can make a choice between intraoral and extraoral approaches. Although intraoral reduction is generally accepted for symphyseal and parasymphyseal fractures, there is still some debate regarding the use of the intraoral approach to condylar neck and subcondylar fractures because of the surgical skills and associated hardware required [5]. Although various methods of external approaches to the mandible have been proposed, there are no recent articles addressing the classic Risdon approach [6-11]. The authors have used the Risdon approach for open reduction and internal fixation of condylar fractures, in some cases combined with external threaded Kirschner wire fixation and rubber traction. The aim of this study was to determine the efficacy and safety of surgical treatment of condylar fractures using the Risdon approach, as well as to describe our clinical experience.

## Methods

A retrospective review of the electronic charts of mandibular condyle neck and subcondylar fractures was performed for the period from March 2008 to June 2012 in the Department of Plastic and Reconstructive Surgery at Hanyang University Guri Hospital. Approval for the study was obtained from the institutional review board on human subjects research and the ethics committee, Hanyang University Guri Hospital (IRB No. 2011–033). Written informed consent for participation in the study was obtained from the participants or their parents if they were children. Mandibular condyle fractures were classified according to the height of the fracture. Mandibular neck fractures occur below the joint capsule attachment but above the sigmoid notch, and subcondyle fractures run from the sigmoid notch to the back edge of the mandibular ramus [12]. According to the relationship between the proximal and the distal segments, the degree of condylar fracture is classified into non-displaced, deviated, or displaced fracture. A non-displaced fracture has no displacement of the fracture site, a deviated fracture is where fracture segments are displaced but some of them contact, and a displaced fracture is where the fracture fragments are separated and the proximal and distal segments do not contact each other. Considering the relationship between the proximal segment and the temporal fossa, a dislocated fracture is one in which the condylar head is deviated from the temporal fossa [12].

### Surgical techniques

Using gentian violet, an incision line was marked 2–3 cm below the lower mandible border, between the angle and the facial notch of the mandible. The incision was normally 4–5 cm, but was extended in either direction in cases of inadequate exposure. After skin incision, dissection was carried out down to the platysma muscle. The muscle was bisected using blunt scissors, and the cervical fascia was cut with care not to damage the facial nerve, until the masseter muscle was exposed. The masseter was cut just above the lower mandible border and dissection was carried out to the periosteum. Reduction was done by the use of wire traction inserted into a drilled hole at the inferior border of the angle. The masseter muscle attached to the posterior border of the mandible ramus was dissected until adequate exposure for reduction was achieved. All patients underwent intermaxillary fixation using arch bars, and the upper and lower jaws were fixed with elastics.

Notably, we decided to operate on condylar neck fracture in children when there was extensive dislocation and no contact between the proximal and the distal fracture segments. In cases of condylar neck fracture in children, a threaded Kirschner wire was placed in the fractured segment percutaneously, and directly in view through the Risdon incision, the condyle head was reduced maximally. The end was then cut to an appropriate length and bent to form a hook. A rubber band was placed on the hook, and the threaded K-wire was pulled in the appropriate direction and fixed with a tongue depressor on a cup to maintain tension. This procedure was performed according to a technique previously described by the authors [13].

## Results

A total of 25 patients, 19 males and 6 females, underwent open reduction via the Risdon approach (Table 1). There were 14 condylar neck fractures and 11 subcondylar fractures. The 14 patients with condylar neck fracture included 11 adults and 3 children. Fifteen cases were caused by traffic accidents, 7 cases by physical altercations, and 3 cases due to industrial accidents. When they were classified according to the degree of condylar fracture, 9 had deviated fractures, 16 had displaced fractures, and 13 had dislocated fractures (Table 2). All 25 patients presented with trismus, 6 with malocclusion, and 3 with open bite. In all of the subcondylar fractures, open reduction and rigid fixation were completed under direct vision. Seven cases of 11 adults with condylar neck fractures were possible to fixate by use of a trochar and 3 condylar neck fractures in children were reduced by external threaded Kirschner wire fixation and rubber traction (Table 3). The result was normal occlusion after surgery. In 3 cases of condylar neck fractures, a slight open bite on the injured side developed after the operation, but ultimately normal occlusion was recovered within several days. The maximal postoperative interincisal distance was 38–56 mm (mean 46.6 mm). There were no malunions, nonunions, avascular necrosis, or ankylosis in the TMJ revealed during the 8-month follow up, which is the average follow-up period. Mild transient neuropraxia of the marginal mandibular nerve was seen in 4 patients, but resolved within 1–2 months.

**Table 1:** The patients’ age and sex

**Age**	**Sex**	
	**Male**	**Female**
1-10	2	
11-20	5	1
21-30	5	
31-40	3	2
41-50	1	3
51-60	2	
61-70	1	

**Table 2:** The etiology and the level of fractures

**Etiology**	**Subcondyle**				**Condyle neck**			
	**Deviated**	**Displaced**	**Displaced, dislocated**	**Deviated, dislocated**	**Deviated**	**Displaced**	**Displaced, dislocated**	**Deviated, dislocated**
TA	2	3			1		7	2
Violence	2	1					1	
Slip or fall	1						2	
IA	1	1					1	

*TA* Traffic accident, *IA* Industrial accident.

**Table 3:** The surgical methods according to the fracture types

**Surgical methods**	**Subcondyle**		**Condyle neck**		
	**Deviated**	**Displaced**	**Deviated**	**Displaced, dislocated**	**Deviated, dislocated**
Risdon approach	6	5	1	3	
Risdon approach + Trochar				6	1
Risdon approach + Kirschner wire				3	

### Case 1

**Figure 1:**
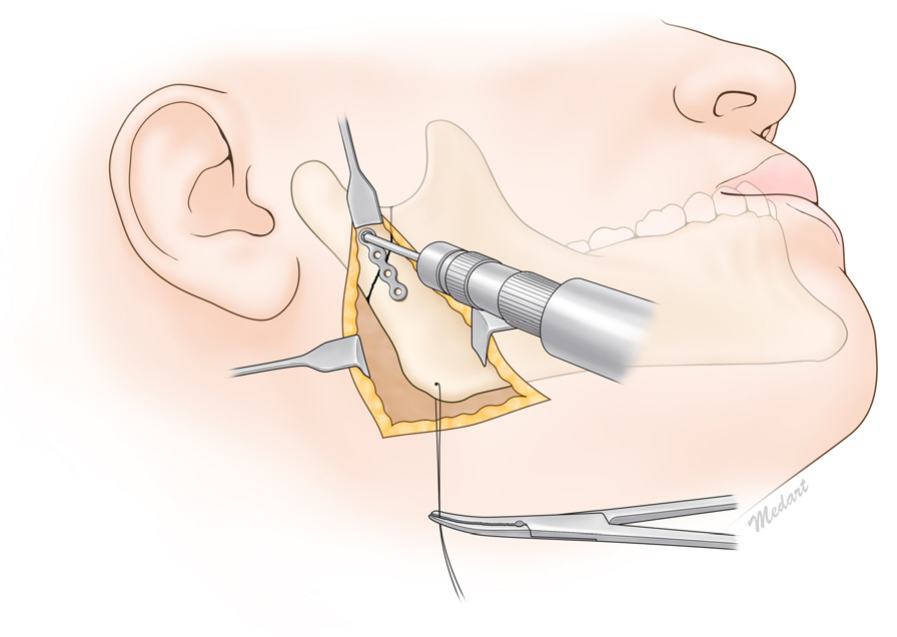
Intraoperative illustrated view of reduction of the subcondylar fracture with the Risdon approach.

**Figure 2:**
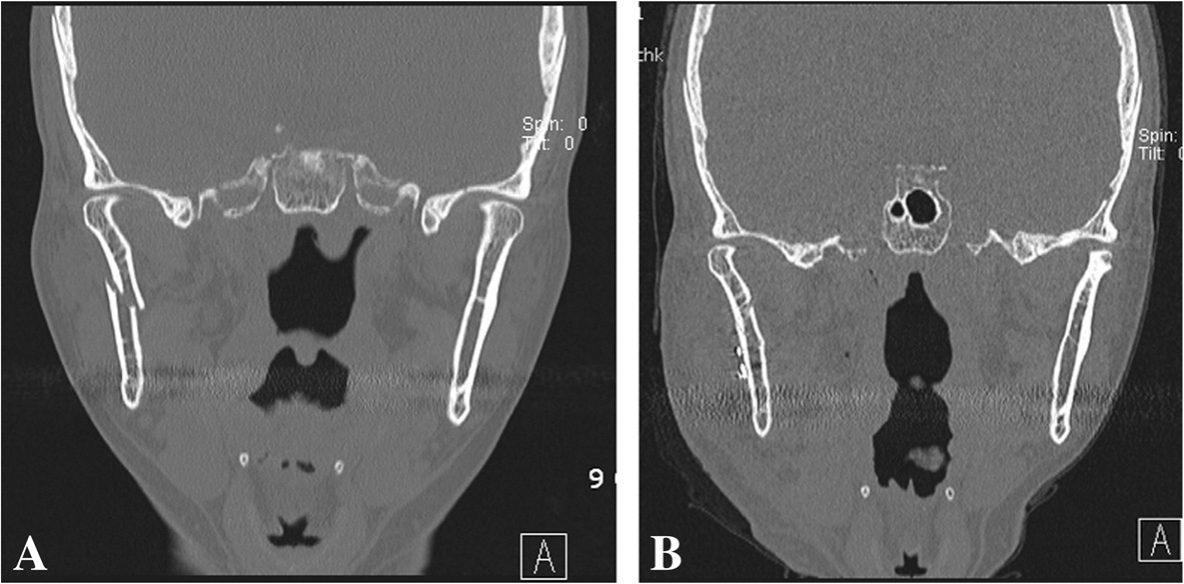
**A 48-year-old female patient with a right subcondylar fracture. A**. The fragment was displaced in the medial direction with the head pointing in the lateral direction. **B**. Reduction and placement of a miniplate to fix the fracture was performed through the Risdon approach.

A 48-year-old female patient visited the outpatient clinic for swelling of the right cheek and difficulty opening her mouth after falling down a set of stairs. The mouth opening was measured to be 15 mm, and the patient showed malocclusion. The right subcondylar fracture was seen on CT images. Intermaxillary fixation with the arch bar method was applied immediately after admission, under local anesthesia. After 5 days, the swelling had subsided dramatically, and reduction was performed under general anesthesia. Using the Risdon incision mentioned above on the right side, the right subcondylar area was exposed. An I-shaped 2.0-mm titanium miniplate was applied on the fracture site after reduction (Figure 1). After fixation, a negative suction drain was placed, and the incision was closed in layers. The patient had normal occlusion on postoperative examination, and elastic bands were applied to immobilize the jaw. The patient was discharged after a week, and the arch bar was removed after 2 weeks during follow-up in the outpatient clinic. The patient was followed up for 9 months, and no complications were observed (Figure 2).

### Case 2

**Figure 3:**
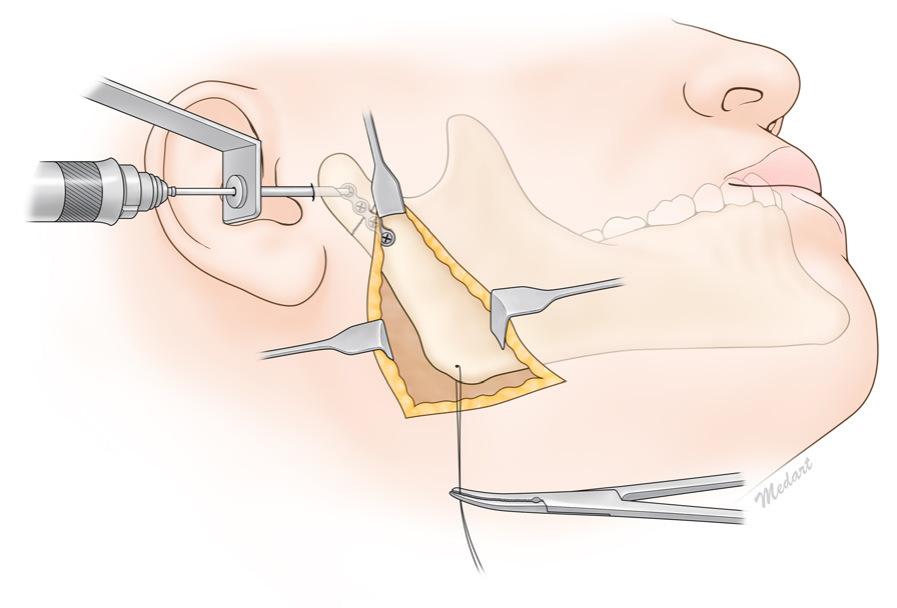
Intraoperative illustrated view of reduction of the condylar neck fracture with the Risdon approach and a trochar.

**Figure 4:**
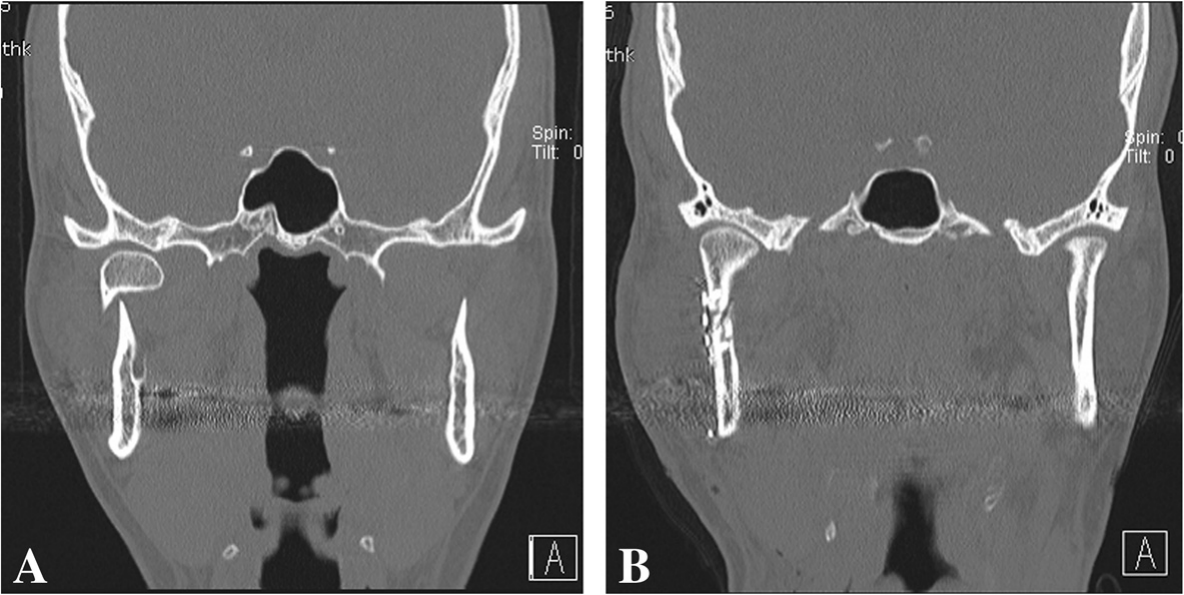
**A 33-year-old male patient with a right condylar neck fracture. A**. Preoperative CT findings. **B**. Reduction and fixation of miniplates was performed with the use of a trochar combined with the Risdon approach.

A 33-year-old male patient visited the emergency room for swelling of the right cheek and difficulty opening his mouth after a slip and fall. Fracture of the right condylar neck was seen on CT images. Intermaxillary fixation with the arch bar method was applied immediately after admission, under local anesthesia. After 7 days, using the Risdon incision method mentioned above on the right side, the right condylar neck was exposed. Two I-shaped 2.0-mm titanium miniplates were affixed to the fracture site, and screw fixation on the fractured condylar neck was performed by using a trochar (Figure 3). The patient had a slight open bite on intraoperative examination, but he had normal occlusion within two days after surgery. The patient was discharged after one week, and the arch bar was removed after 2 weeks during follow-up in the outpatient clinic. The patient was followed up for 7 months, and no complications were observed (Figure 4).

### Case 3

**Figure 5:**
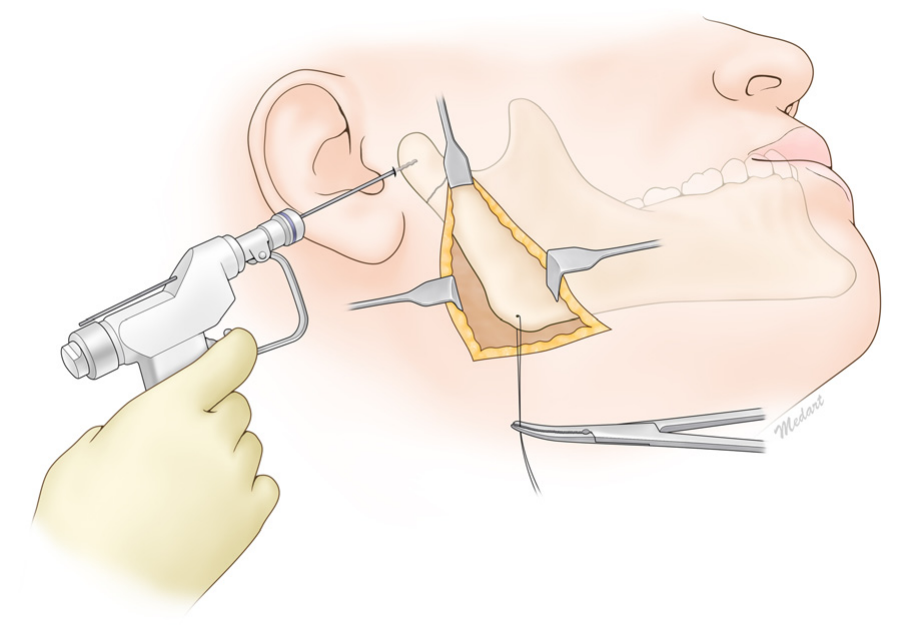
Intraoperative illustrated view of reduction of the condylar neck fracture with the Risdon approach and a Kirschner wire.

**Figure 6:**
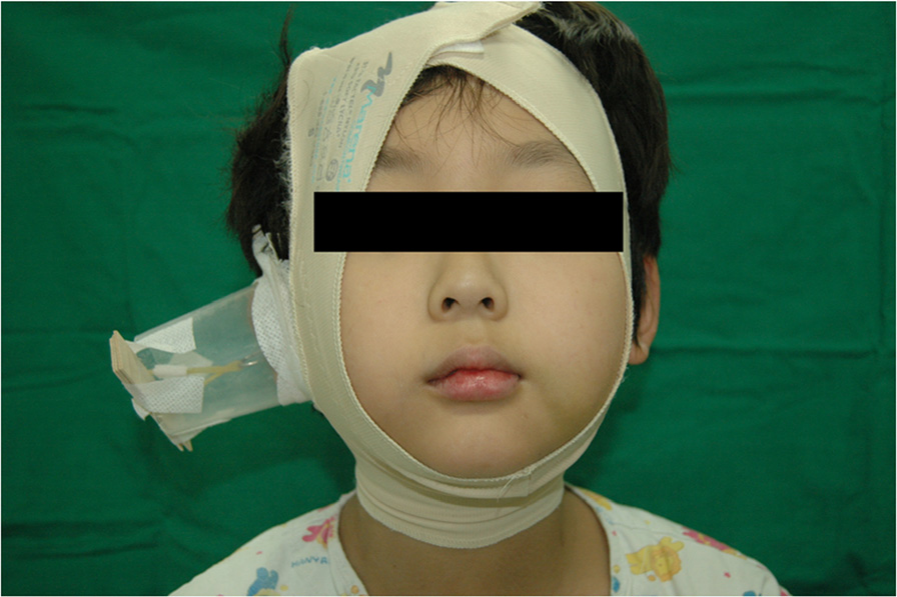
**Clinical photograph of a patient with traction on the threaded Kirschner wire with rubber bands.** The rubber bands were tied to a tongue depressor, which was then fixed to a cup placed on the patient’s cheek.

**Figure 7:**
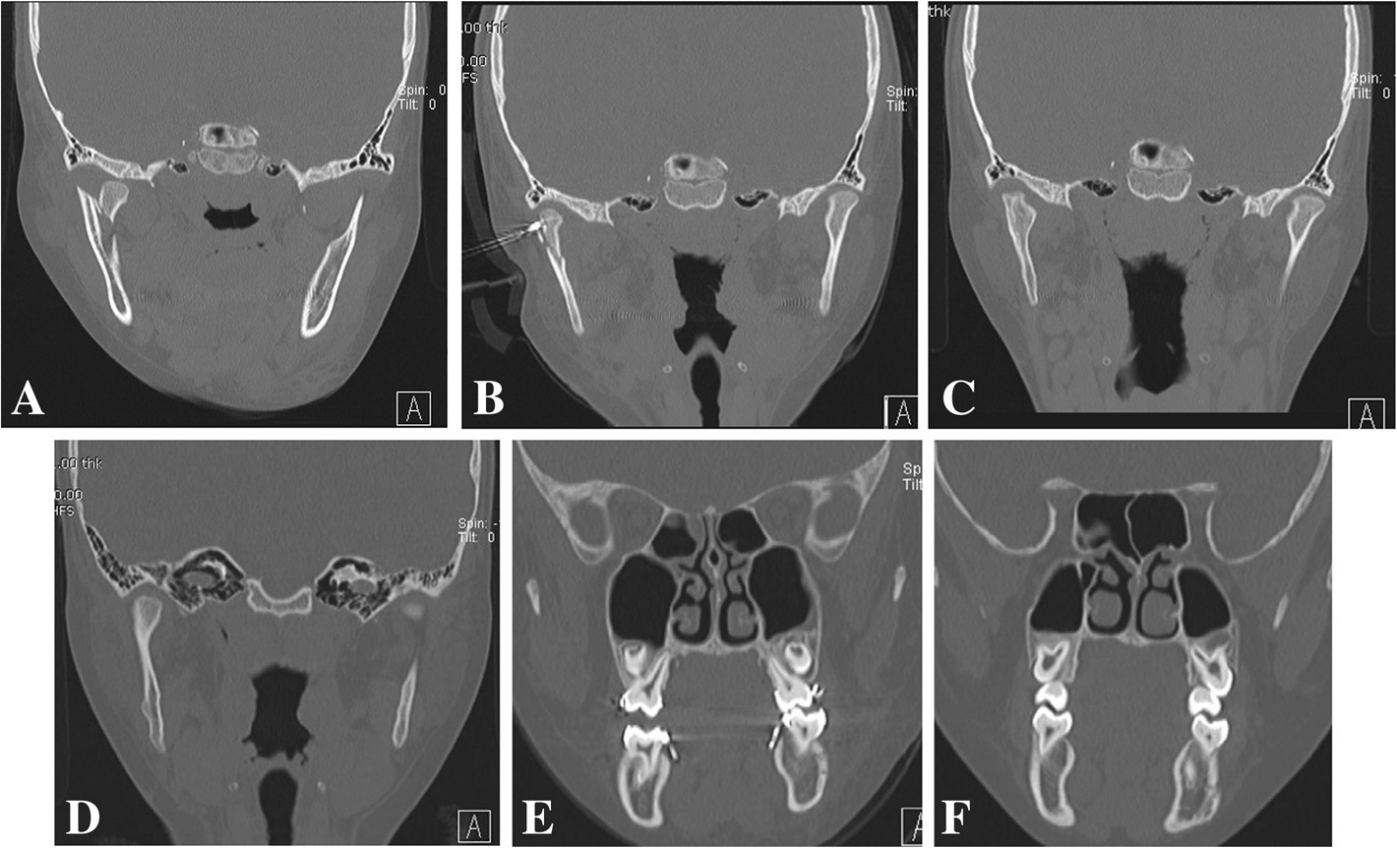
**CT image of the fracture site in a pediatric patient with a condylar neck fracture. A**. The fractured segment was displaced and dislocated in the medial direction and the proximal and the distal segments had no contact with each other. **B**. Percutaneous threaded Kirschner wires were placed in the condylar fracture segment under direct vision through the Risdon approach. The fractured segment was reduced to its original position. **C**. CT findings six months postoperatively. **D**. CT findings two years postoperatively show excellent union of the condylar neck with the normally shaped condylar head. **E**. CT findings preoperatively show an open bite on the injured side.** F**. CT findings postoperatively show normal occlusion.

**Figure 8:**
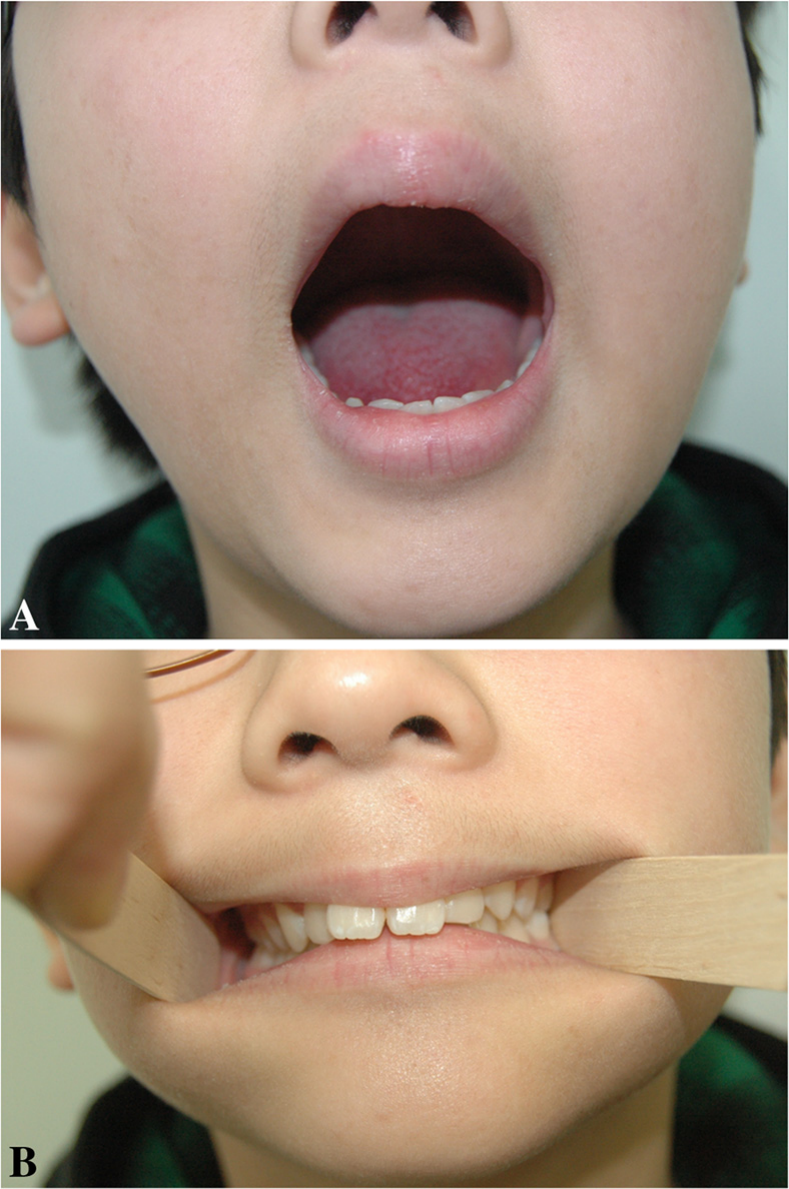
**Photograph of the same patient two years postoperatively. A**. Facial palsy was not observed and full symmetrical mouth opening was possible. Interincisal distance was found to be 40 mm. **B**. The patient had neutro-occlusion postoperatively.

A 10-year-old male patient visited the emergency room after an automobile accident. The right TMJ region was painful, and the patient had an open bite. Mouth opening was measured at 10 mm. Fractures of the left angle and right condylar neck were seen on CT images. We decided to operate on the condylar neck fracture because the degree of condylar neck fracture was extensive dislocation without contact between the fracture segments. The patient was admitted to our department since there were no other comorbidities. In the operating room, intermaxillary fixation was perfomed using the Erlich arch bar method. The Risdon incisions were made using the techniques mentioned above, and fixation of the left angle was performed using absorbable miniplates and screws after reduction. For the right condylar neck, the Risdon approach was used to expose the fracture site. After making a 0.5 cm stab incision at the preauricular area, a threaded Kirschner wire was inserted percutaneously into the fractured segment under direct vision at the incision. After threaded K-wire placement, a hole was drilled at the inferior border of the right mandibular angle, and wire traction was applied to the angle inferiorly, while the segment was reduced to its original position (Figure 5). The external end of the threaded K-wire was bent, and using an elastic band, constant traction was applied by the use of a tongue depressor fixed to a plastic cup put on the right cheek (Figure 6). All incisions were closed in layers after a negative suction drain was inserted. The arch bar and the threaded K-wire were removed after 2 and 3 weeks, respectively, under local anesthesia. The patient has received follow-up care for 24 months, and no complications have been observed (Figure 7). He was allowed full use of the TMJ and normal occlusion (Figure 8).

## Discussion

Various approaches have been described in the literature for the reduction of mandibular condyle fractures. The intraoral approach has the advantages of avoiding leaving a scar on the face, simultaneous control of the occlusion and repositioning of the fragments during the operation, and direct visualization of the occlusion during placement of the hardware. However, the intraoral approach requires special traction, lighting devices to better expose the fracture site, and more surgical time than the extraoral approach [5]. Recently, an endoscopy-assisted approach has been widely reported in the literature. Besides requiring specialized instruments, surgeons need additional training to use endoscopic equipment and there is a surgical learning curve because the techniques involve indirect incision without allowing for extensive exposure [14].

Extraoral approaches to the mandibular condyle region can be divided into three major categories in terms of the height of the approach: high, middle and low [15]. The high approach, involving the preauricular and perilobular approach, has been applied to the reduction of high condylar neck or condylar head fractures. These approaches have a very well camouflaged scar and achieve a much clearer and more direct exposure than the middle and low approaches. However, this technique requires the identification of the facial nerve trunk or its branches, at least two at a time, to allow for surgical access between these branches. This requires advanced dissection skills and confidence in the anatomy of the facial nerves in the buccal and mandibular region [10,11]. Despite meticulous dissection, mild neuropraxia can persist up to 13 months postoperatively, and in cases of a transparotid approach, postoperative sialoceles and salivary fistulas can be a nuisance [3].

Middle height approaches to the mandible include the retromandibular approach. This approach allows better exposure of the mandibular condyle compared to the low approach. However, this approach involves identification of the buccal and marginal mandibular branches of the facial nerve so as to avoid possible facial nerve damage. Despite this careful identification of the facial nerve, this approach requires retraction of the parotid gland, which may lead to facial nerve injury [2].

**Figure 9:**
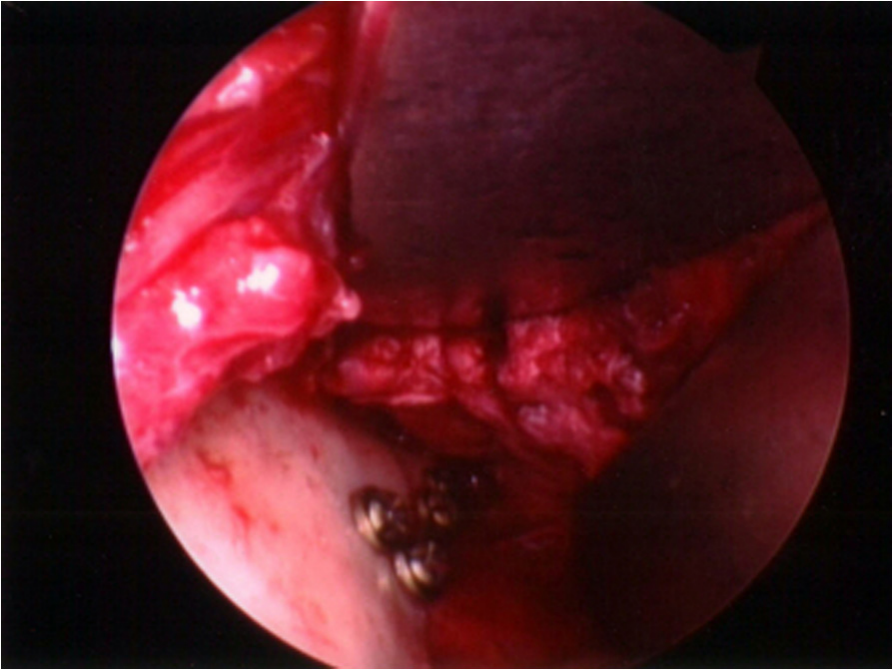
**Intraoperative view of subcondylar fracture via the Risdon approach.** Adequate exposure of the surgical field can be achieved with sufficient dissection of the masseter muscle.

One low height approach to the mandible is the Risdon approach. Approaching the mandible from an incision below the marginal mandibular nerve is the most crucial point in the Risdon approach. The marginal mandibular nerve is identified easily without much dissection, and if a flap is elevated, including the nerve, there is no risk of facial nerve damage. In the retromandibular, high submandibular, or periangular approaches, which are similar to each other, dissecting between the buccal and marginal mandibular nerves can be difficult for inexperienced hands, even though anatomic studies performed by the authors have shown there is no risk of nerve injury [8,9]. The Risdon approach is easily learned and performed, requiring virtually no learning curve. Identifica-tion of the marginal mandibular branch of the facial nerve is very simple, and there are ways to elevate the skin muscle flap without the need to identify the facial nerve [6]. However, it is known that this approach allows less exposure of the mandibular ramus and condyle, and most surgeons would agree that this approach insufficiently exposes condylar fractures. However, in our experience, this approach has allowed for direct visualization, reduction, and fixation of all subcondylar lesions through the ample detachment of the masseter muscle attached to the posterior border of the mandibular ramus (Figure 9). Moreover, when open reduction and fixation is impossible with only the Risdon approach for higher lesions such as condylar neck fracture, reduction could be performed well by using a trochar combined with the Risdon approach.

In children with condylar neck fracture, only percutaneous threaded K-wire fixation and rubber traction without rigid fixation produced good results because children have a high capacity for bony union. Moreover, a threaded K-wire can easily be removed under local anesthesia after bony union. We previously reported on the percutaneous manipulation of condylar fractures under fluoroscopy [13]. The fractured condylar segment can be manipulated by threaded K-wires inserted percutaneously under fluoroscopy, and with rubber traction during the postoperative period, reduction is well maintained. However, closed reduction using the threaded K-wire has a steep learning curve, and since the fractured segment is small, precise insertion of the threaded K-wire into the fractured segment is necessary for reduction so as not to comminute the segment. In our experience, inserting the threaded K-wire under direct vision through the Risdon approach allows for a smaller failure rate and a more exact reduction than under fluoroscopic vision alone.

**Figure 10:**
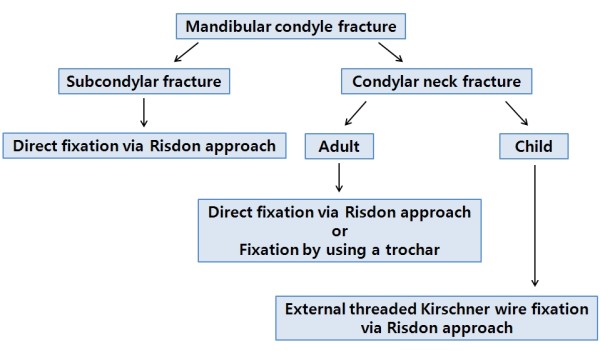
Algorithm of treatment for mandibular condyle fractures using the Risdon approach.

Therefore, we propose that reduction and fixation of subcondylar fractures under direct vision are possible by the Risdon approach. In condylar neck fractures, the use of a trochar in adults and threaded K-wire fixation and rubber traction in children aided in the satisfactory reduction and fixation of the fractured segment (Figure 10).

## Conclusions

In an era where novel and modified approaches are proliferating, it may be concluded that the Risdon approach is one good surgical approach to the reduction of mandibular condyle fractures on the basis of these clinical results.

## Informed consent

Written informed consent was obtained from the patient or his parent if a patient is a child for publication of cases of this study and any accompanying images. A copy of written consent is available for review by the Editor-in-Chief of this journal.

## Competing interests

The authors declare that they have no competing interests.

## Authors’ contributions

SM N and JH L performed the design of the study and drafted the manuscript. JH K carried out data acquisition and helped to draft the manuscript. All authors read and approved the final manuscript.

## Pre-publication history

The pre-publication history for this paper can be accessed here:

http://www.biomedcentral.com/1471-2482/13/25/prepub

## References

[B1] Choi K Yang J Chung H Cho B Current concepts in the mandibular condyle fracture management Part II: Open reduction versus closed reduction Arch Plast Surg 2012 39 301 308 10.5999/aps.2012.39.4.301 22872831 PMC3408273

[B2] Manisali M Amin M Aghabeigi B Newman L Retromandibular approach to the mandibular condyle: a clinical and cadaveric study Int J Oral Maxillofac Surg 2003 32 253 256 10.1054/ijom.2002.0270 12767870

[B3] Vesnaver A Gorjanc M Eberlinc A Dovsak DA Kansky AA The periauricular transparotid approach for open reduction and internal fixation of condylar fractures J Craniomaxillofac Surg 2005 33 169 179 10.1016/j.jcms.2005.01.008 15878517

[B4] Zachariakes N Mezitis M Mourouzis C Papadakis D Spanou A Fractures of the mandibular condyle: a review of 466 cases. Literature review, reflections on treatment and proposals J Craniomaxillofac Surg 2006 34 421 432 10.1016/j.jcms.2006.07.854 17055280

[B5] Toma VS Mathog RH Toma RS Meleca RJ Transoral versus extraoral reduction of mandible fractures: a comparison of complication rates and other factors Otolaryngol Head Neck Surg 2003 128 215 219 10.1067/mhn.2003.59 12601317

[B6] Kanno T Mitsugi M Sukegawa S Fujioka M Furuki Y Submandibular approach through the submandibular gland fascia for treating mandibular fractures without identifying the facial nerve J Trauma 2010 68 641 643 10.1097/TA.0b013e31819ea15f 19797989

[B7] Girotto R Mancini P Balercia P The retromandibular transparotid approach: Our clinical experience J Craniomaxillofac Surg 2012 40 78 81 10.1016/j.jcms.2011.01.009 21306910

[B8] Bhavsar D Barkdull G Berger J Tenenhaus M A novel surgical approach to subcondylar fractures of mandible J Craniofac Surg 2008 19 496 499 10.1097/SCS.0b013e3181539b8b 18362731

[B9] Lutz JC Clavert P Wolfram-gabel R Wilk A Kahn JL Is the high submandibular transmasseteric approach to the mandibular condyle safe for the inferior buccal branch? Surg Radiol Anat 2010 32 963 969 10.1007/s00276-010-0663-z 20461515

[B10] Baek RM Min KH Heo CY Eun SC The perilobule approach to subcondylar fractures Ann Plast Surg 2011 66 253 256 10.1097/SAP.0b013e3181e1333f 21042178

[B11] Özkan HS Sahin B Gorgu M Melikoglu C Results of transmasseteric anteroparotid approach for mandibular condylar fractures J Craniofac Surg 2010 21 1882 1883 10.1097/SCS.0b013e3181f4aef7 21119445

[B12] Choi K Yang J Chung H Cho B Current concepts in the mandibular condyle fracture management Part I: Overview of condylar fracture Arch Plast Surg 2012 39 291 300 10.5999/aps.2012.39.4.291 22872830 PMC3408272

[B13] Kim JH Nam DH Kwon I Ahn HS Lee YM The treatment for mandibular condyle fracture of children by a threaded Kirschner wire and external rubber traction J Korean Soc Plast Reconstr Surg 2009 36 221 224

[B14] Kellman RM Cienfuegos R Endoscopic approaches to subcondylar fractures of the mandible Facial Plast Surg 2009 25 23 28 10.1055/s-0028-1112228 19206025

[B15] Knepil GJ Kanatas AN Loukota RJ Classification of surgical approaches to the mandibular condyle Br J Oral Maxillofac Surg 2011 49 664 665 10.1016/j.bjoms.2011.02.009 21453998

